# Individual and contextual factors associated with orofacial dysfunction in schoolchindren

**DOI:** 10.1590/0103-644020256228

**Published:** 2025-04-14

**Authors:** Luiza Jordânia Serafim de Araújo, Monalisa Cesarino Gomes, Ramon Targino Firmino, Edja Maria Melo de Brito Costa, Saul Martins de Paiva, Ana Flávia Granville-Garcia

**Affiliations:** 1 Department of Dentistry, School of Dentistry, Universidade Estadual da Paraíba, Campina Grande, PB, Brazil; 2 Department of Dentistry, Centro Universitário UNIFACISA, Campina Grande, PB, Brazil; 3 Academic Unit of Biological Sciences, Universidade Federal de Campina Grande, Patos, PB, Brazil.; 4 Department of Pediatric Dentistry, Federal University of Minas Gerais, Minas Gerais, PB, Brazil.

**Keywords:** Stomatognathic system, anxiety, malocclusion, dental caries, family relations

## Abstract

Investigar fatores individuais e contextuais associados à disfunção orofacial em escolares. Foi realizado um estudo transversal com 739 crianças de oito a dez anos de idade. As crianças responderam a questionários abordando disfunção orofacial e ansiedade. Os responsáveis ​​forneceram informações sobre características sociodemográficas, distúrbios do sono e coesão familiar. Os examinadores investigaram a presença de disfunção orofacial, cárie dentária, má oclusão e lesões dentárias traumáticas (critérios de Andreasen) (Kappa>0,80). O tipo de escola e a renda média mensal do bairro escolar foram as variáveis ​​contextuais. Estatística descritiva foi realizada para caracterizar a amostra e modelos de regressão de Poisson multinível não ajustados e ajustados (p <0,05) foram executados. A prevalência de disfunção orofacial foi de 33,3%. Após ajuste pelas variáveis ​​contextuais, menor renda familiar, maior quantidade de dentes cariados, presença de lesão dentária traumática, má oclusão grave/muito grave, presença de distúrbios do sono e ansiedade permaneceram associados à disfunção orofacial, enquanto má oclusão definida foi um fator de proteção. Em termos de contexto, frequentar escola pública foi associado à disfunção orofacial. A disfunção orofacial foi influenciada por menor renda familiar, maior quantidade de dentes cariados e presença de lesão dentária traumática, distúrbios do sono e ansiedade. Além disso, frequentar escola pública foi o determinante contextual que desempenhou um papel significativo no resultado.

## Abstract (en)

To investigate individual and contextual factors associated with orofacial dysfunction in schoolchildren. A cross-sectional study was conducted with 739 children eight to ten years of age. The children answered questionnaires addressing orofacial dysfunction and anxiety. Guardians provided information on sociodemographic characteristics, sleep disturbances, and family cohesion. Examiners investigated the presence of orofacial dysfunction, dental caries, malocclusion, and traumatic dental injuries (Andreasen criteria) (Kappa>0.80). The type of school and average monthly income of the school neighborhood were the contextual variables. Descriptive statistics were performed to characterize the sample. Unadjusted and adjusted (p <0.05) multilevel Poisson regression models were run. The prevalence of orofacial dysfunction was 33.3%. After adjustment by the contextual variables, a lower family income, a larger quantity of carious teeth, the presence of traumatic dental injury, severe/very severe malocclusion, the presence of sleep disturbances, and anxiety remained associated with orofacial dysfunction, whereas definite malocclusion was a protection factor. In terms of context, attending a public school was associated with orofacial dysfunction. Orofacial dysfunction was influenced by a lower family income, a larger quantity of carious teeth, and the presence of traumatic dental injury, sleep disturbances, and anxiety. Moreover, attending a public school was the contextual determinant that played a significant role in the outcome.

**Figure:**
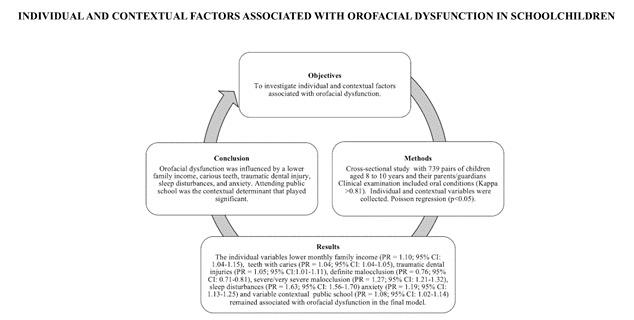

## Introduction

Orofacial function plays an essential role in interpersonal relations ^1^. When impaired, the stomatognathic system can be affected, leading to orofacial dysfunction ^1^, which is a highly prevalent condition, affecting 87.6% of children eight to ten years of age ^2^. Orofacial dysfunction can compromise the performance of daily activities and cause symptoms, such as pain, limited jaw movements, and chewing difficulties ^3^.

Oral conditions, such as dental caries, malocclusion, and traumatic dental injuries, are common among children with orofacial dysfunction and exert an important influence on chewing and swallowing ^4^ ^,^ ^5^ ^,^ ^6^. Thus, oral health status can interfere with orofacial function in children. Moreover, this age group is characterized by an environment involving daily activities that can increase anxiety and favor the emergence of muscle tension in the face ^7^. Thus, anxiety may be associated with orofacial dysfunction ^8^. However, no studies have investigated this association in children eight to ten years of age.

The prevalence of sleep disorders in children is reported to be 8.58% ^9^. Such disorders can cause cognitive dysfunction, stunted growth, and changes in daytime habits ^10^. Moreover, an association has been reported between poor sleep quality and orofacial dysfunction ^9^. Thus, changes in sleep patterns can exert a relevant influence on orofacial functions.

Family cohesion is defined as the degree of dependence and connection among family members and is another essential factor that can impact the oral health of children. A possible association has been reported between families with low cohesion and the presence of harmful oral habits in children ^11^. Thus, it is necessary to study the effect of family cohesion on orofacial dysfunction.

Contextual determinants of the school setting can exert an influence, as schoolchildren spend a large part of the day in this space ^12^. Moreover, public schools tend to have fewer health promotion activities ^12^. Thus, school-related factors, such as the type of school and the average income of the surrounding neighborhood, may influence the occurrence of orofacial dysfunction.

The multilevel approach and the investigation of contextual determinants can reveal the influence of variables overlooked in studies that did not adopt such an approach. This enables new lines of investigation for the determination of causal relationships in childhood. Therefore, the present study aimed to investigate individual and contextual factors associated with orofacial dysfunction in schoolchildren. The null hypothesis is that individual factors, such as anxiety, do not exert an influence on the occurrence of orofacial dysfunction.

## Materials and methods

### Ethical approval statement

This study received approval from the institutional Human Research Ethics Committee (approval nº 3.255.174; certificate of presentation: 10514619.2.0000.5187) by Resolution nº 466/2012 of the National Board of Health and followed the ethical principles established in the Declaration of Helsinki (2013). The children signed a term of assent and a statement of informed consent was signed by a legal guardian.

### Study design and sample

A cross-sectional study was developed with 739 schoolchildren eight to ten years of age enrolled in public and private schools in the city of Campina Grande, Brazil. The sample was obtained through a probabilistic sampling procedure in two stages (schools and children) involving weighting and adjustment for the design effect. In the first stage, 14 public schools and 18 private schools distributed among the six geographical regions of the city were randomly selected. The minimum sample size was calculated considering the number of public and private schools in each geographical region. In the second stage, participants were randomly selected from each school through a simple random drawing based on attendance lists. This process resulted in a sample comprising 520 students from public schools and 249 from private schools. The sample size was calculated for an analytical study involving the comparison of two proportions using the G*Power program, version 3.1.9.7 (Franz Faul, Universitat Kiel, Germany), considering a 95% significance level and 95% study power. Based on the pilot study, the proportion of orofacial dysfunction among adolescents with and without anxiety was 80% and 60%, respectively. Data on this variable provided the largest sample to investigate the association of interest. The minimum sample was determined to be 286 adolescents, to which a design effect of 2.0 was applied and 25% was added to compensate for possible dropouts, resulting in a target sample of 763 adolescents. 

### Elegibility criteria

Schoolchildren eight to ten years of age enrolled in public and private schools in the city of Campina Grande, Brazil, were included. Children with developmental problems and cognitive deficiency reported by parents/guardians, and those having been submitted to orthodontic treatment were excluded.

### Training and calibration exercises

Training and calibration were conducted in two steps. In the theoretical phase, a specialist trained four dentists in the diagnosis of orofacial dysfunction using the Nordic Orofacial Test-Screening (NOT-S), malocclusion using the Dental Aesthetic Index (DAI), dental caries using the International Caries Detection and Assessment System (ICDAS), and traumatic dental injuries using the criteria proposed by Andreasen et al. (2007). In the practical step, the examiners diagnosed oral problems in 40 children (20 from a public school and 20 from a private school) chosen by convenience. A specialist in orthodontics and pediatric dentistry led the calibration exercise and served as the “gold standard” for the determination of inter-examiner agreement. The children were examined a second time after a seven-day interval for the determination of intra-examiner agreement. The calibration exercise involved the determination of the Kappa statistic, which revealed a good level of agreement among the examiners (K> 0.80). 

### Pilot study

A pilot study was conducted with 40 children selected following the same eligibility criteria as the main study. The results showed no need to alter the methods. The children in the pilot study were not included in the main study.

### Clinical data collection

The children performed supervised brushing to improve visibility during the diagnosis and were examined in a reserved room at the schools standing in front of the examiner, who wore personal protective equipment and an LED headlamp. The examination was performed with the aid of a mouth mirror (PRISMA, SP, Brazil), Williams probe (WHO-621), and gauze to dry the teeth. At the end of the examination, topical fluoride was applied to children with active caries, and the children were given information on their oral health status. A letter was also sent to the parents/guardians with counseling and referral to a primary care unit or teaching clinic of a public dental school, when necessary.

Orofacial dysfunction was investigated using the Nordic Orofacial Test-Screening. The NOT-S has 12 domains, with data collected from an interview (six domains) and clinical examination (six domains). The interview addresses sensory function, breathing, habits, salivation, chewing, swallowing, and dry mouth sensations. The clinical examination assesses breathing, face at rest, facial expressions, masticatory muscles, and other jaw functions as well as oral motor function and speech. Each domain is scored 1 when impaired and 0 when not impaired. The NOT-S total score ranges from 0 to 12. Orofacial dysfunction was recorded when at least four domains had either a ‘yes’ answer or impaired function. This instrument has been validated and adapted to Brazilian Portuguese for use on individuals eight to 14 years of age ^13^.

Malocclusion was diagnosed using the Dental Aesthetic Index (DAI), which is a quantitative index recommended by the World Health Organization for assessing the aesthetic impact of the occlusion. The DAI results in a classification that identifies the degree of malocclusion severity and orthodontic treatment need: normal occlusion with no treatment need (DAI ≤25); definite malocclusion suggesting elective treatment (DAI 26-30); severe/very severe malocclusion with treatment highly recommended (DAI≥ 31) ^(^ ^14^.

Dental caries was diagnosed using the International Caries Detection and Assessment System (ICDAS), which scores carious lesions based on extent: 0 - sound; 1 - visible change after drying; 2 - visible change in color without drying; 3 - decayed enamel; 4 - dark shadow underlying dentin; 5 - cavity with dentin exposed at base; and 6 - extensive cavity with dentin exposed at base and on walls. Scores “1” and “2” were grouped into code 2, as it was not possible to dry the teeth with compressed air. The number of teeth with carious lesions was recorded ^15^.

Traumatic dental injuries were investigated based on the classification proposed by Andreasen et al. (2007): enamel fracture, enamel + dentin fracture, complicated crown fracture, extrusive luxation, lateral luxation, intrusive luxation, avulsion, discoloration, and restoration due to injury. Traumatic dental injury was recorded if any of these categories were present ^(^ ^16^.

### Non-clinical data collection

The children were approached in the classroom and an explanation was given on how data collection would be performed. The statement of informed consent, sociodemographic questionnaire, Sleep Disturbance Scale for Children, and Family Adaptability and Cohesion Evaluation Scales were distributed to the children to be completed by a parent/caregiver at home.

The sociodemographic questionnaire addressed objective items related to the parents/guardians (monthly family income and level of schooling) and items concerning the child (sex and age). These variables were collected as continuous data and dichotomized by the median.

Sleep disturbances were investigated with the aid of the Sleep Disturbance Scale for Children between three and 18 years of age. This scale is composed of 26 items addressing the child’s sleep habits in the previous six months. The sum of the item scores furnishes a total score ranging from 26 to 130 points. In the present study, a cutoff point of 39 was considered indicative of the presence of sleep disturbance. This instrument has been validated and adapted for use in children in Brazil ^17^.

The Family Adaptability and Cohesion Evaluation Scales (FACES III) comprise 20 items, the scores of which determine the degree of family cohesion and adaptability. Families are classified into four groups: disengaged, with a high degree of independence among the members (10-34); separated, with a certain degree of independence among the members (35-40); connected, with moderate dependence among the members (41-45); and enmeshed, with a higher degree of dependence among the members (46-50) ^(^ ^18^.

Children also answered a questionnaire addressing anxiety, which was investigated using the Revised Children’s Manifest Anxiety Scale for individuals eight to 13 years of age. This scale is comprised of 37 items separated into two subscales: an anxiety subscale and a ‘lie’ subscale. The anxiety index was determined by the total sum of items with a ‘yes’ answer. The score ranges from 0 to 37 points, with higher scores denoting a greater anxiety trait. Only the anxiety subscale was used in the present study. This instrument has been validated and adapted for use in children in Brazil. For statistical purposes, the anxiety score was dichotomized (absent or present) based on the cutoff point according to sex: 10.68 ± 5.32 for boys and 14.01 ± 5.57 for girls ^19^.

Two variables were used to investigate the influence of context on orofacial dysfunction: type of school (public or private), collected on the first visit to the school, and average monthly income of the school neighborhood, dichotomized by the median.

### Statistical analysis

Data analysis was performed with the aid of SPSS Statistics (SPSS for Windows, version 25.0; IBM Inc., Armonk, NY, USA). Descriptive statistics were performed for the characterization of the sample. Unadjusted and adjusted multilevel Poisson regression models were run to determine associations between the outcome and predictors. Multilevel Poisson regression analysis involved a random-effects model with random intercepts and fixed slopes to determine associations between orofacial dysfunction and the individual and contextual covariables. This strategy enabled estimates of prevalence ratios (PR) between comparison groups and respective 95% confidence intervals (CI).

In the first step, an unconditional (null) model was used to estimate variability in the data before the incorporation of individual and contextual characteristics. The second model (Model 2) included covariables on the individual level and the third model (Model 3) included both individual and contextual covariables. Variables with a p-value <0.20 in the bivariate multilevel Poisson regression were incorporated first and those with a p-value <0.05 were maintained in Model 2. Next, contextual variables with a p-value <0.20 were incorporated into the bivariate multilevel Poisson regression, and those with a p-value <0.05 remained in Model 3 (the final model). The goodness of fit of the models was calculated based on deviance values (-2 log-likelihood). Multicollinearity among the variables was also checked. 

## Results

The final sample was composed of 739 (96%) children and their parents/guardians. Missing data occurred due to absences of children on three consecutive visits to the school and refusals to participate in the study (3.9%) (Table 1).

The prevalence of orofacial dysfunction was 33.3%. The prevalence of dental caries, traumatic dental injuries, severe/very severe malocclusion, and definite malocclusion was 72.0%, 16.2%, 29.1%, and 20%, respectively. Moreover, sleep disturbance and anxiety were found in 58.9% and 18.1% of the sample, respectively (Table 1).

**Table 1:** Characterization of the sample.

Variables	n (%)
Outcome variable	
Orofacial dysfunction	
No	493 (66.7)
Yes	246 (33.3)
Variables on an individual level	
Sex	
Male	370 (50.1)
Female	369 (49.9)
Monthly family income	
≤ US$ 235.84	327 (57.0)
> US$ 235.84	247 (43.0)
Guardian’s schooling	
≤ 8 years of study	318 (43.1)
> 8 years of study	419 (56.9)
Dental caries	
No	207 (28.0)
Yes	532 (72.0)
Sleep disturbance	
No	435 (58.9)
Yes	304 (41.1)
Traumatic dental injury	
No	619 (83.8)
Yes	120 (16.2)
Degree of malocclusion severity	
Severe/Very Severe	215 (29.1)
Definite	148 (20.0)
Normal	376 (50.9)
Anxiety	
No	605 (81.9)
Yes	134 (18.1)
Family cohesion	
Disengaged	242 (32.7)
Separated	314 (42.5)
Connected	152 (20.6)
Enmeshed	31 (4.2)
Variables on a contextual level	
Type of school	
Public	349 (47.2)
Private	390 (52.8)
Monthly income of school neighborhood	
≤ US$ 283.30	318 (43.0)
> US$ 283.30	421 (57.0)

In the bivariate multilevel Poisson regression analysis, significant associations were found with the following variables: lower monthly family income (PR = 1.42; 95% CI: 1.02-1.97), lower schooling of the guardian (PR = 1.32; 95% CI: 1.01-1.73), larger quantity of teeth with caries (PR = 1.05; 95% CI: 1.03-1.08), traumatic dental injuries (PR = 1.37; 95% CI: 0.99-1.87), severe/very severe malocclusion (PR = 1.51; 95% CI: 1.21-1.89), definite malocclusion (PR = 1.15; 95% CI: 0.86-1.52), sleep disturbance (PR = 1.68; 95% CI: 1.25-2.27), and anxiety (PR = 1.60; 95% CI: 1.20-2.13). In terms of context, attending a public school was associated with orofacial dysfunction (PR =1.47; 95% CI: 1.11-1.96) (Table 2).

**Table 2:** Bivariate analysis of factors associated with orofacial dysfunction considering individual and contextual levels in children eight to ten years of age.

	Orofacial dysfunction		Bivariate	
			(unadjusted PR†)	
Variable	Present n (%)	Absent n (%)	p-value	95% CI
Variables on an individual level				
Sex				
Male	120 (32.4)	250 (67.6)		1.00
Female	126 (34.1)	243 (65.9)	0.297	1.15 (0.88-1.52)
Monthly family income				
≤ US$ 235.84	126 (38.5)	201 (61.5)	0.033*	1.42 (1.02-1.97)
> US$ 235.84	70 (28.3)	177 (71.7)		1.00
Guardian’s schooling				
≤ 8 years of study	124 (39.0)	194 (61.0)	0.042	1.32 (1.01-1.73)
> 8 years of study	122 (29.1)	297 (70.9)		1.00
Number of teeth with caries (mean/SD)	4.35 (3.97)	3.12 (3.38)	0.000*	1.05 (1.03-1.08)
Traumatic dental injury				
No	198 (32.0)	421 (68.0)		1.00
Yes	48 (40.0)	72 (60.0)	0.051*	1.37 (0.99-1.87)
Degree of malocclusion severity				
Severe/Very Severe	92 (42.8)	123 (57.2)	0.000*	1.51 (1.21 -1.89)
Definite	48 (32.4)	100 (67.6)	0.332	1.15 (0.86 -1.52)
Normal	106 (28.2)	270 (71.8)		1.00
Sleep disturbance				
No	73 (24.0)	231 (76.0)		1.00
Yes	173 (39.8)	262 (60.2)	0.001*	1.68 (1.25-2.27)
Family cohesion				
Disengaged	95 (39.3)	147 (60.7)	0.401	1.39 (0.64-3.04)
Separated	106 (33.8)	208 (66.2)	0.689	1.17 (0.53-2.56)
Connected	38 (25.0)	114 (75.0)	0.999	1.00 (0.43-2.29)
Enmeshed	7 (22.6)	24 (77.4)		1.00
Anxiety				
No	183 (30.2)	422 (69.8)		1.00
Yes	63 (47.0)	71 (53.0)	0.001*	1.60 (1.20-2.13)
Variables on a contextual level				
Type of school				
Public	135 (38.7)	214 (61.3)	0.007*	1.47 (1.11-1.96)
Private	111 (28.5)	279 (71.5)		1.00
Monthly income of school neighborhood				
≤ US$ 283.30	113 (35.5)	205 (64.5)	0.373	1.12 (0.86-1.47)
> US$ 283.30	133 (31.6)	288 (68.4)		1.00

†Unadjusted rate ratios (RR) for multilevel binomial regression to determine associations between individual/contextual variables and orofacial dysfunction among school children.

*Variables included in the multivariate model (p <0.20).

CI: confidence interval

The results of the multivariate multilevel Poisson regression analysis are displayed in Table 3. After adjustments by the contextual variables (Model 3), a lower monthly family income (PR = 1.10; 95% CI: 1.04-1.15), a larger quantity of teeth with caries (PR = 1.04; 95% CI: 1.04-1.05), presence of traumatic dental injuries (PR = 1.05; 95% CI:1.01-1.11), definite malocclusion (PR = 0.76; 95% CI: 0.71-0.81), severe/very severe malocclusion (PR = 1.27; 95% CI: 1.21-1.32), sleep disturbances (PR = 1.63; 95% CI: 1.56-1.70), and anxiety (PR = 1.19; 95% CI: 1.13-1.25) remained associated with orofacial dysfunction. In terms of context, attending a public school remained associated with the outcome (PR = 1.08; 95% CI: 1.02-1.14) (Table 3).

**Table 3:** Multilevel Poisson regression for orofacial dysfunction considering individual and contextual levels in children eight to ten years of age.

	Orofacial dysfunction		
	Model 1 (“Null”)	Model 2	Model 3
Fixed effects		PR (95% CI)	PR (95% CI)
Intercept	0.31 (0.31-0.32)	0.16 (0.15-0.16)	0.15 (0.15-0.16)
Variables on an individual level			
Monthly family income			
≤ US$ 235.84	-	1.13 (1.08-1.18)	1.10 (1.04-1.15)
> US$ 235.84	-	1.00	1.00
Guardian’s schooling			
≤ 8 years of study	-	1.04 (1.01-1.09)	-
> 8 years of study	-	1.00	-
Number of teeth with caries		1.05 (1.04-1.06)	1.04 (1.04-1.05)
Traumatic dental injury			
No	-	1.00	1.00
Yes	-	1.06 (1.01-1.11)	1.05 (1.01-1.11)
Degree of malocclusion severity			
Severe/Very Severe	-	1.27 (1.22-1.33)	1.27 (1.21-1.32)
Definite		0.76 (0.71-0.80)	0.76 (0.71-0.81)
Normal		1.00	1.00
Sleep disturbance			
No	-	1.00	1.00
Yes	-	1.63 (1.56-1.71)	1.63 (1.56-1.70)
Anxiety			
No	-	1.00	1.00
Yes	-	1.19 (1.14-1.25)	1.19 (1.13-1.25)
Variables on a contextual level			
Type of school			
Public	-	-	1.08 (1.02-1.14)
Private	-	-	1.00
Random effects			
Deviance (-2 log-likelihood)	54,658.224	41,605.940	41,598.390

Model 1 (“null”): unconditional model; Model 2: individual variables; Model 3: variables on individual and contextual levels.

CI: confidence interval

## Discussion

In the present study, a lower monthly family income, a greater quantity of teeth with caries, traumatic dental injuries, malocclusion severity, sleep disturbances, and anxiety remained associated with orofacial dysfunction. The contextual factor ‘type of school’ also exerted a significant influence on the outcome. Thus, the hypothesis of the study was confirmed, as anxiety influenced orofacial dysfunction. This study is relevant, as it emphasizes a contextual analysis of the situation of the school and describes associations between individual/contextual factors and orofacial dysfunction in childhood.

The prevalence of orofacial dysfunction was 33.3%. A previous study found that 87.8% of children had an affected Nordic Orofacial Test-Screening domain ^2^. This divergence may have occurred due to socioeconomic standards, as regions with better economic standards and higher educational levels may be more likely to identify cases of orofacial dysfunction. These rates underscore the need for the early detection of this condition to enable treatment and adequate follow-up, avoiding the occurrence of functional repercussions in adulthood.

A larger quantity of teeth with caries was associated with orofacial dysfunction. Carious lesions can cause pain and discomfort in the oral cavity, leading to inadequate compensatory habits and affecting vital functions, such as chewing and speaking ^20^. The study cited also reported that dental caries were associated with the prevalence of orofacial dysfunction ^20^. Thus, monitoring is recommended for the diagnosis and early treatment of dental caries to prevent its impact on orofacial function.

Very severe malocclusion remained associated with orofacial dysfunction. Such oral problems can interfere with chewing function and speech ^(^ ^21^. A previous study found a correlation between malocclusions, such as crossbite and functional lateral deviation, and orofacial dysfunction, as these problems cause functional difficulties and masticatory instability ^(^ ^1^ ^,^ ^4^. In contrast, malocclusion with a lower degree of severity served as a protection factor, possibly due to causing less of an impact on oral function, since malocclusion of lower severity can be self-corrected during the transition to permanent dentition ^22^. Thus, early intervention in cases of severe malocclusion is necessary to correct the defect and prevent orofacial dysfunction.

Traumatic dental injuries also negatively influence orofacial dysfunction. Such incidents can directly damage the teeth, interfere with normal occlusion, and exert a negative impact on masticatory function, causing pain and tooth sensitivity. A previous study conducted with children found that traumatic dental injuries contributed to functional problems, with a negative impact on quality of life ^23^. Treatment should be prompt to relieve pain and discomfort, to restore orofacial function in children.

An association was found between sleep disturbances and orofacial dysfunction. This was likely due to behavioral and physiological factors related to inadequate sleep, such as grinding the teeth, which leads to an imbalance in the muscles of the face, causing facial pain and strain on the masticatory system ^24^. A previous study also found a positive correlation between sleep disorders and orofacial dysfunction in children ^9^. Therefore, controlling sleep habits through an established routine and an adequate environment and improving lifestyle habits could reduce sleep disturbances and possibly minimize the occurrence of orofacial dysfunction.

The data collected in the present study confirmed the hypothesis that anxiety exerts an influence on orofacial dysfunction. This is explained by the fact that children are unable to control their emotions and can have greater muscle tension in the orofacial region, possibly due to childhood being a period permeated with academic burdens and expectations, contributing to the occurrence of headaches and facial pain ^7^. A previous study found that anxiety influenced orofacial dysfunction in adolescents aged 10 to 14 years ^8^. Thus, children should be under the care of healthcare providers to identify and control anxiety, thus reducing its consequences.

A lower family income exerted an influence on the occurrence of orofacial dysfunction, possibly because lower income families have limited access to resources and dental services. A systematic review also reported low family income to be a risk factor for orofacial dysfunction ^25^. This underscores the importance of studying and identifying health disparities to reduce rates of chronic diseases in the population.

Contextual factors related to the school environment, such as type of school, also influenced orofacial dysfunction, possibly due to the fact that resources are limited at public schools, which offer less oral health support to children and have fewer health promotion actions ^12^. Thus, children at public schools are negatively affected in terms of self-care and hygiene, which compromises oral health. Schools should promote awareness and ensure egalitarian access to dental services.

The present study has limitations related to the cross-sectional design, which impedes the establishment of cause-and-effect relationships. Longitudinal studies are needed to monitor these children for a longer period, observing possible changes and establishing causal relationships. However, this study also has strong points, such as the representative sample of children, lending external validity to the results, the conduction of a pilot study to test the methods, the fact that the examiners underwent training and calibration exercises, and the use of validated instruments, thus minimizing the risk of bias.

The establishment of actions directed at orofacial dysfunction in childhood is necessary, as this problem can be aggravated with age when not treated ^(^ ^2^ and can impair speech, swallowing, nasal breathing, and masticatory function ^4^. An early diagnosis enables the adoption of preventive actions to avoid or minimize the consequences of this condition. Moreover, the use of low-cost assessment tools to screen for orofacial dysfunction constitutes a strategy for minimizing difficulties in gaining access to dental services and directing efforts at more vulnerable groups. The high prevalence of orofacial dysfunction reflects the need for public policies with a multidisciplinary approach and early interventions. These aspects can positively impact the development of oral functions and, consequently, enhance quality of life.

## Conclusion

In conclusion, the results of this study indicate that a lower monthly family income, greater quantity of teeth with caries, traumatic dental injuries, malocclusion, sleep disturbances, and anxiety exerted an influence on the occurrence of orofacial dysfunction. With regards to contextual determinants, attending a public school played a significant role in the outcome.
